# Genome characteristics of atypical porcine pestivirus from abortion cases in Shandong Province, China

**DOI:** 10.1186/s12985-023-02247-0

**Published:** 2023-11-29

**Authors:** Xiaoyu Sun, Qiaoya Zhang, Hu Shan, Zhi Cao, Juan Huang

**Affiliations:** 1 College of Veterinary Medicine, Qingdao Agricultural University, Qingdao, 266109 China; 2 Shandong Collaborative Innovation Center for Development of Veterinary Pharmaceuticals, Qingdao, China; 3 Qingdao Research Center for Veterinary Biological Engineering and Technology, Qingdao, China

**Keywords:** Atypical porcine pestivirus (APPV), Viral metagenomics, Abortion cases, Phylogenetic analysis

## Abstract

**Background:**

Atypical porcine pestivirus (APPV) is a novel, highly variable porcine pestivirus. Previous reports have suggested that the virus is associated with congenital tremor (CT) type A-II in piglets, and little information is available about the correlation between the virus and sow abortion, or on coinfection with other viruses. In China, reported APPV strains were mainly isolated from South China and Central China, and data about the APPV genome from northern China are relatively scarce.

**Methods:**

Eleven umbilical cords, one placenta, and one aborted piglet, were collected from aborted sows of the same farm in Shandong Province of northern China. Nucleic acids were extracted from the above samples, and subsequently pooled for viral metagenomics sequencing and bioinformatics analysis. The viral coexistence status and complete genome characteristics of APPV in Shandong Province were determined.

**Results:**

In abortion cases, APPV was present with Getah virus, porcine picobirnavirus, porcine kobuvirus, porcine sapovirus, Po-Circo-like virus, porcine serum-associated circular virus, porcine bocavirus 1, porcine parvovirus 1, porcine parvovirus 3 and porcine circovirus 3, etc. The first complete genome sequence(11,556 nt) of APPV in Shandong Province of northern China, was obtained using viral metagenomics and designated APPV-SDHY-2022. Comparison with Chinese reference strains revealed that the polyprotein of APPV-SDHY-2022 shared 82.6-84.2%, 93.2-93.6%, and 80.7-85% nucleotide identity and 91.4-92.4%, 96.4-97.7%, and 90.6-92.2% amino acid identity with those of the Clade I, Clade II and Clade III strains, respectively. Phylogenetic analysis based on the complete polyprotein CDS and NS5A sequences concluded that APPV-SDHY-2022 belongs to Clade II. Analysis of the NS5A nucleotide sequences revealed homology of greater than 94.6% for the same isoform, 84.7-94.5% for different isoforms of the same clade and 76.8-81.1% for different clades. Therefore, Clade II was further divided into three subclades, and APPV-SDHY-2022 belonged to subclade 2.3. Members of Clade II have 20 unique amino acids in individual proteins, distinguishing them from Clade I and Clade III members. The E2 protein showed the greatest diversity of putative N-glycosylation sites with 9 patterns, and APPV-SDHY-2022 along with other Chinese APPV strains shared the conserved B-cell conformational epitope residues 39E, 70R, 173R, 190K and 191N of the E2 protein.

**Conclusions:**

We reported viral coexistence and the first complete genome sequence of APPV from abortion cases and from Shandong Province. The new APPV isolate belongs to an independent branch of Clade II. Our results increase the molecular and epidemiological understanding of APPV in China.

**Supplementary Information:**

The online version contains supplementary material available at 10.1186/s12985-023-02247-0.

## Background

Atypical porcine pestivirus (APPV) belongs to the genus *Pestivirus* in the family *Flaviviridae* and is a novel, highly differentiated pestivirus that was first identified in pigs in the USA through metagenomic sequencing in 2015 [1]. APPV was classified as Pestivirus K by the International Committee on Taxonomy of Viruses (ICTV) in 2018 [2]. The clinical presentation of pigs infected with APPV is characterized by congenital tremor (CT) type A-II in piglets [3], while adult pigs may become viral carriers and shedders [4]. It is not surprising that APPVs are present and have become a major threat in China, which is an important country for pig farming and trade [5].

APPV is a highly variable single-stranded RNA virus, and its genome is approximately 11.0 kb in size and comprises a single open reading frame (ORF) flanked by untranslated regions (UTRs) at the 5’- and 3’-ends. The ORF encodes a continuous polyprotein, which is processed into 12 mature proteins, including four structural proteins (C, E^rns^, E1, and E2) and eight nonstructural proteins (N^pro^, P7, NS2, NS3, NS4A, NS4B, NS5A, and NS5B) [1]. The NS3 gene has been shown to be highly conserved in Chinese strains of APPV, while the NS5A, N^pro^ and E^rns^ genes are highly variable [6]. All of the Chinese strains can be classified into 3 genotypes (clades) and 5 subgenotypes (subclades) (1.2 and 1.4–1.7) within genotype 1 [6]. The genomic presence of APPV has been detected in pigs from southern China, including Guangdong, Guangxi, Guizhou, Jiangxi, Yunnan, Anhui and other provinces [7–14]. However, the presence of APPV strains in pigs from northern China is rare to date.

Previous studies on APPV in China were conducted in CT cases, and investigations on the presence of APPV and coinfection with other viruses in abortion cases are still scarce. Methods commonly used for RNA virus detection include RT–PCR, real-time PCR (qRT–PCR), PCR cloning for Sanger sequencing and in situ hybridization [15]. However, traditional detection methods are limited in that the genome sequence of the virus has to be known before detection. In contrast, next-generation sequencing (NGS) has provided new technologies to circumvent some of the challenges of targeted RNA sequencing. Platforms such as Illumina allow next-generation sequencing (NGS) of novel viruses without targets, which is important for uncovering new pathogens, detecting combinations of infections and diagnosing complex cases [16].

In this study, viral coexistence was determined, and the first complete genome sequence of APPV in Shandong Province of northern China was acquired from abortion cases using viral metagenomics after failure to detect abortion-related pathogens such as Brucella, porcine reproductive and respiratory syndrome virus (PRRSV), porcine parvovirus 1(PPV1), classical swine fever virus (CSFV) and porcine circovirus 2(PCV2) by PCR or RT–PCR. Our results contribute to the epidemiological investigation and comprehensive understanding of APPV in China.

## Materials and methods

### Ethics statement

This study was approved by the Animal Ethical & Welfare Committee of the College of Veterinary Medicine, Qingdao Agricultural University, Qingdao, China.

### Sample preparation

In May 2022, an unexplained abortion occurred at a farm in Haiyang, Shandong Province, and the sows had no other symptoms of infection or disease. Thirteen abortion samples, namely, eleven umbilical cords, one placenta, and one aborted piglet, were collected from the farm. The above samples (0.5 g each) were weighed out in a 1.5 mL centrifuge tube, ground using a grinder and then diluted by adding 200 μL of DPBS (Solarbio) to each tube and stored at -80 °C. The samples were subjected to three freeze-thaw cycles and were then centrifuged at 12 000× *g* for 3 min at 4 °C. The supernatants of all samples were pooled together and filtered using a 0.45 μm filter (Millipore) and concentrated using an ultrafiltration tube (Millipore). The concentrated samples were digested using micrococcal nuclease (New England Biolabs) for two hours at 37 °C. At the end of the reaction, EGTA (Solarbio; 500 mM, pH = 8) was added to inactivate the micrococcal nuclease. DNA and RNA were then extracted separately using a nucleic acid extraction kit (Magen). Next, the extracted nucleic acids were subjected to sequencing.

### Viral metagenomic analysis

In this study, the extracted RNA and DNA were sequenced using Illumina sequencing technology, and an Illumina paired-end (PE) library was constructed. The sequencing data obtained were quality-controlled by removing low-quality data and contaminated sequences including rRNA, host and bacterial sequences. After quality control, Kraken 2 software was selected for sequence comparison, and species with a relative abundance higher than 1% were selected as candidates for further analysis. The clean reads obtained above were de novo-assembled using SPAdes and SOAPdenovo software. The contigs obtained from the above assembled results were compared and evaluated with the virus-NT database in BLAST (V2.10.0+). The candidate reference sequences and the species with the closest evolutionary relationship were determined. The assembled scaffold files were subjected to in-depth statistical analyses, i.e., the determination of the degree of read coverage in the spliced contigs to verify the sequencing accuracy and read coverage relative to the splicing results. Contigs ≥ 1500 bp in length were selected for in-depth statistical analysis.

### APPV confirmation by NS3 gene RT–PCR and sequencing

To confirm the existence of APPV in samples and verify the correctness of the NGS results, a pair of primers (Forward: 5’ - CTCACCAGTGATGGGTGGGA − 3’,

Reverse: 5’ - CCTATTTTTTTCATGAACACCATGGC − 3’) was designed according to the NS3 gene sequence of APPV-SDHY-2022. RT–PCR amplification was performed using a one-step RT–PCR kit (TaKaRa) according to the manufacturer’s instructions. The amplification was conducted in a 50 μL volume as follows: 50 °C for 30 min and 94 °C for 2 min for the RT reaction, followed by 35 cycles of amplification at 94 °C for 30 s, 56 °C for 30 s, and 72 °C for 30 s, with a final extension at 72 °C for 7 min. The 440 bp-length PCR products were subjected to Sanger sequencing (Sangon Biotech), and the obtained sequence was assembled and compared with that of the NS3 gene in APPV-SDHY-2022 using MegAlign software.

### APPV genetic evolutionary analysis

**Fig. 1:**
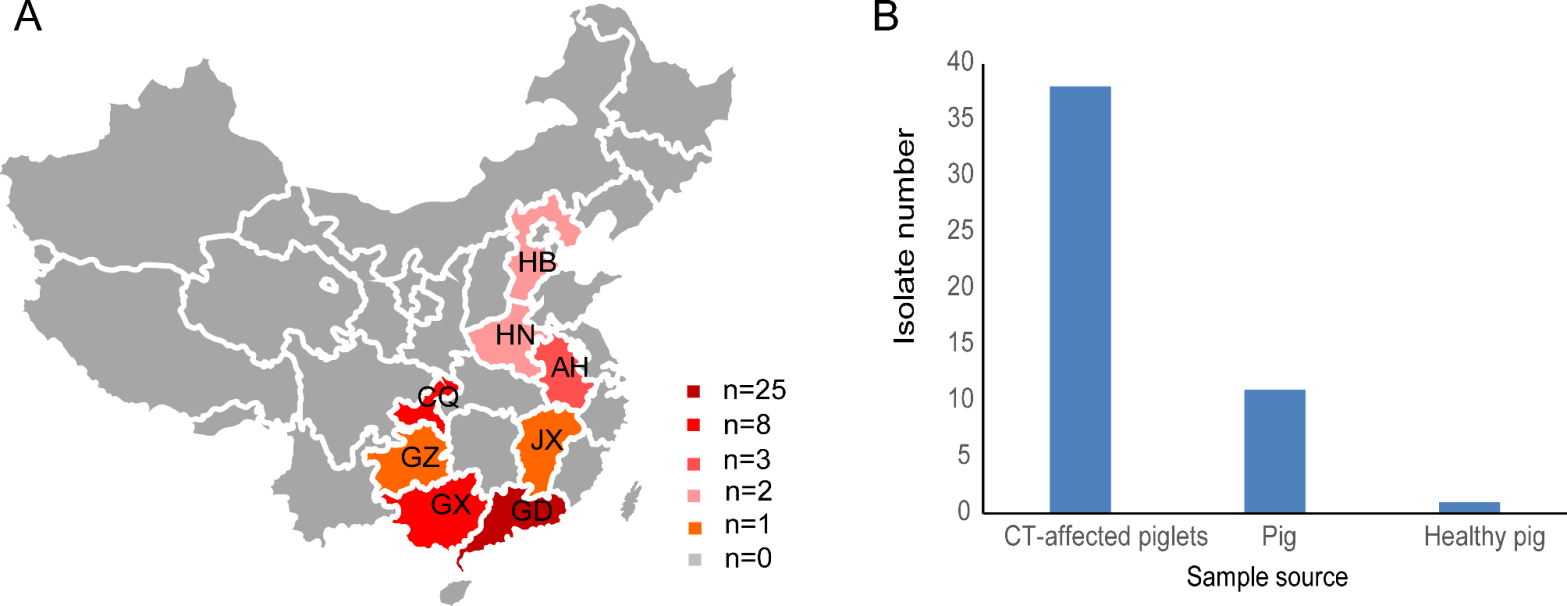
Information on the Chinese reference strains used in this study. (**A**) Geographic distribution of the APPV strains with complete CDSs or genome sequences from China. GD, Guangdong; GX, Guangxi; GZ, Guizhou; JX, Jiangxi; CQ, Chongqing; AH, Anhui; HN, Henan; HB, Hebei. (**B**) The sample source of the APPV strains with complete CDSs or genome sequences from China

The complete genomic sequence of APPV was determined from this study and designated APPV-SDHY-2022. Fifty coding sequences (CDSs) of polyproteins from Chinese reference strains downloaded from the GenBank database were used for multiple sequence alignment analysis by MegAlign software with the Clustal W method. Detailed information on these reference sequences, including province/region, collection dates, strain names, accession numbers, sample source, sample type, gene type and references [4, 7, 9, 11–14, 17–21], was included in Additional file 1: Table S1. The geographical distribution and sample source of the APPV strains with complete CDS or genome sequences from China were summarized in Fig. 1. The homology of individual proteins and their corresponding nucleotides was also analyzed. Phylogenetic analysis was performed based on complete polyprotein CDS and NS5A sequences by the neighbor-joining (NJ) method with 1,000 bootstrap replicates in MEGA11 software.

### Recombination, amino acid sequence, glycosylation analysis and antigen prediction

Potential recombination events were identified using Recombination Detection Program version 4 (RDP4) and BootScan and then examined using SimPlot software version 3.5.1. Amino acid sequences of viral proteins were aligned with reference strains using MEGA11 and BioEdit software. The putative N-glycosylation sites within the E^rns^, E1 and E2 sequences of Chinese APPV strains were predicted according to a glycosylation analysis algorithm (http://www.cbs.dtu.dk/services/). The potential antigenicity of the APPV E2 protein was predicted computationally using the Jameson-Wolf method by the Protean tool of DNAStar software. The potential B-cell conformational epitopes of the E2 protein in APPV Chinese strains were predicted by BepiPred-3.0 ( https://services.healthtech.dtu.dk/service.php?BepiPred-3.0), and residues with a higher score (reaching or crossing the threshold value 0.1512) were more likely to be part of a B-cell epitope [22].

## Results

### Viral metagenomic analysis

The number of clean reads was 21,157,543 for the RNA sample and 26,789,502 for the DNA sample. For RNA, the data were assembled to a total sequence length of 2,337,534, with 60.92% GC content. The length of the largest contig was 11,556 nt, which was identified as APPV (Table 1), and named as APPV-SDHY-2022 for further analysis in this study. For DNA, the data were assembled with a total sequence length of 38,447,346 and 41.71% GC content. Other viruses, including Getah virus, porcine picobirnavirus, porcine kobuvirus, porcine sapovirus, Po-Circo-like virus, porcine serum-associated circular virus, porcine bocavirus 1, porcine parvovirus 1, porcine parvovirus 5 and porcine circovirus 3 were also identified by sequence alignment ((Table 1), however, most contigs of these viruses were less than 500 bp (see Additional file 2: Table s2 & Table s3). No other known pathogens (PRRSV, PPV2-4/6–8, CSFV, PCV2 and Japanese encephalitis virus) related to abortion were sequenced.

**Table 1:** Blast results of assembled sequences from viral metagenomic sequencing*

Sequencing samples	Alignment subjects	Reads counts	Assembled sequences	
RNA	Atypical porcine pestivirus	48	Complete genome	
	Getah virus	28	Partial sequence	
	Porcine kobuvirus	6	Partial sequence	
	Hubei picorna-like virus 61	/	Partial sequence	
	Porcine picobirnavirus	/	Partial sequence	
	Bovine picobirnavirus	/	Partial sequence	
	Human picobirnavirus	/	Partial sequence	
	Simian picobirnavirus	/	Partial sequence	
	Posavirus 3	/	Partial sequence	
	Posavirus 1	/	Partial sequence	
	Porcine sapovirus	/	Partial sequence	
	Mamastrovirus 3	/	Partial sequence	
DNA	Po-Circo-like virus	/	Partial sequence	
	uncultured human fecal virus	/	Partial sequence	
	Porcine serum-associated circular virus	/	Partial sequence	
	Porcine polyomavirus	/	Partial sequence	
	IAS virus	/	Partial sequence	
	Porcine bocavirus 1	/	Partial sequence	
	Porcine parvovirus 1	/	Partial sequence	
	Porcine parvovirus 5	/	Partial sequence	
	Bovine herpesvirus type 1.2	/	Partial sequence	
	Bovine alphaherpesvirus 1	7	Partial sequence	
	Porcine circovirus 3	/	Partial sequence	

* Phage and alignment length less than 100 bp sequences were not included

### APPV confirmation by NS3 gene RT–PCR and sequencing

APPV presence was confirmed in the pooled sample by RT–PCR amplification targeting the NS3 gene (see Additional file 3: Fig.s1A). The assembled sequence of the PCR products was identical to that of APPV-SDHY-2022 (see Additional file 3: Fig.s1B). This provided additional evidence of APPV presence in the abortion cases.

### Genome sequence and homology analysis of APPV

The genome of strain APPV-SDHY-2022 (GenBank accession no. OP381297) contains 11,556 nucleotides (nt) and consists of a 5’UTR (370 nt, positions 1 to 370), CDS (10,909 nt, 371 to 11,279), and 3’UTR (277 nt, 11,280 to 11,556). The nucleotide and amino acid sequences of the individual proteins of the strains were aligned separately, and the homology between APPV-SDHY-2022 and the reference strains was determined (Table 2). Sequence alignment based on APPV polyprotein CDS showed that the nucleotide identities of APPV-SDHY-2022 with Clade I, Clade II, and Clade III strains were 82.6-84.2%, 93.2-93.6%, and 80.7-85%, respectively, while the amino acid identities were 91.4-92.4%, 96.4-97.7%, and 90.6-92.2%, respectively. APPV-SDHY-2022 shared the highest nucleotide identity (93.6%) with APPV-China/GD-SHM/2016, and the highest amino acid identity (97.7%) with GD-YJHSEY2N. Among the 12 mature proteins, NS5A showed the lowest homology (77.6-93.3% at the nt level) with the reference strains.

**Table 2:** Homology analysis of APPV-SDHY-2022 with Chinese reference strains (%)

Regions	Clade I		Clade II		Clade III	
	nt	aa	nt	aa	nt	aa
polyprotein	82.6–84.2	91.4–92.4	93.2–93.6	96.4–97.7	80.7–85	90.6–92.2
N^pro^	78-81.5	79.4–83.3	90.9–92.6	90.6–93.9	77.4–77.8	80-81.1
C	78.4–84.1	83.8–90.1	93.4–95.2	95.5–98.2	80.5–81.4	91–91
E^rns^	80.8–83	89.5–94.3	91.9–94.4	95.7–98.1	80-80.6	92.4–93.3
E1	83.2–85.8	94-97.5	90.2–94	95.5–97.5	80.1–80.7	92.5–94.5
E2	84.1–86.9	91.3–95.9	92.1–93.6	94.2–96.7	80.6–81.2	89.6–92.1
P7	78.1–95.3	78.1–95.8	89.1–93.8	89.6–93.8	76.6–77.6	77.1–78.1
NS2	81.3–89.8	90.1–95.2	91.8–93.8	95.2–96.5	79.7–80.7	88.9–89.8
NS3	84.5–86	95.5–97.2	93.4–94.3	98.4–99.9	83.6–95	96.1–96.7
NS4A	78.6–84.1	89.6–95.5	91–94	97-98.5	78.1–89.1	89.6–91
NS4B	83.2–85.5	95-97.1	92.9–93.7	98.2–99.4	80.1–81.1	94.4–95.3
NS5A	79.1–80.6	84.4–87.1	92.4–93.3	95.6–96.8	77.6–78.3	83.1–83.5
NS5B	82.7–84	90.2–92.2	93.1–93.9	96.5–97.6	80.9–90.4	89.6–93.6

### Phylogenetic analysis

**Fig. 2:**
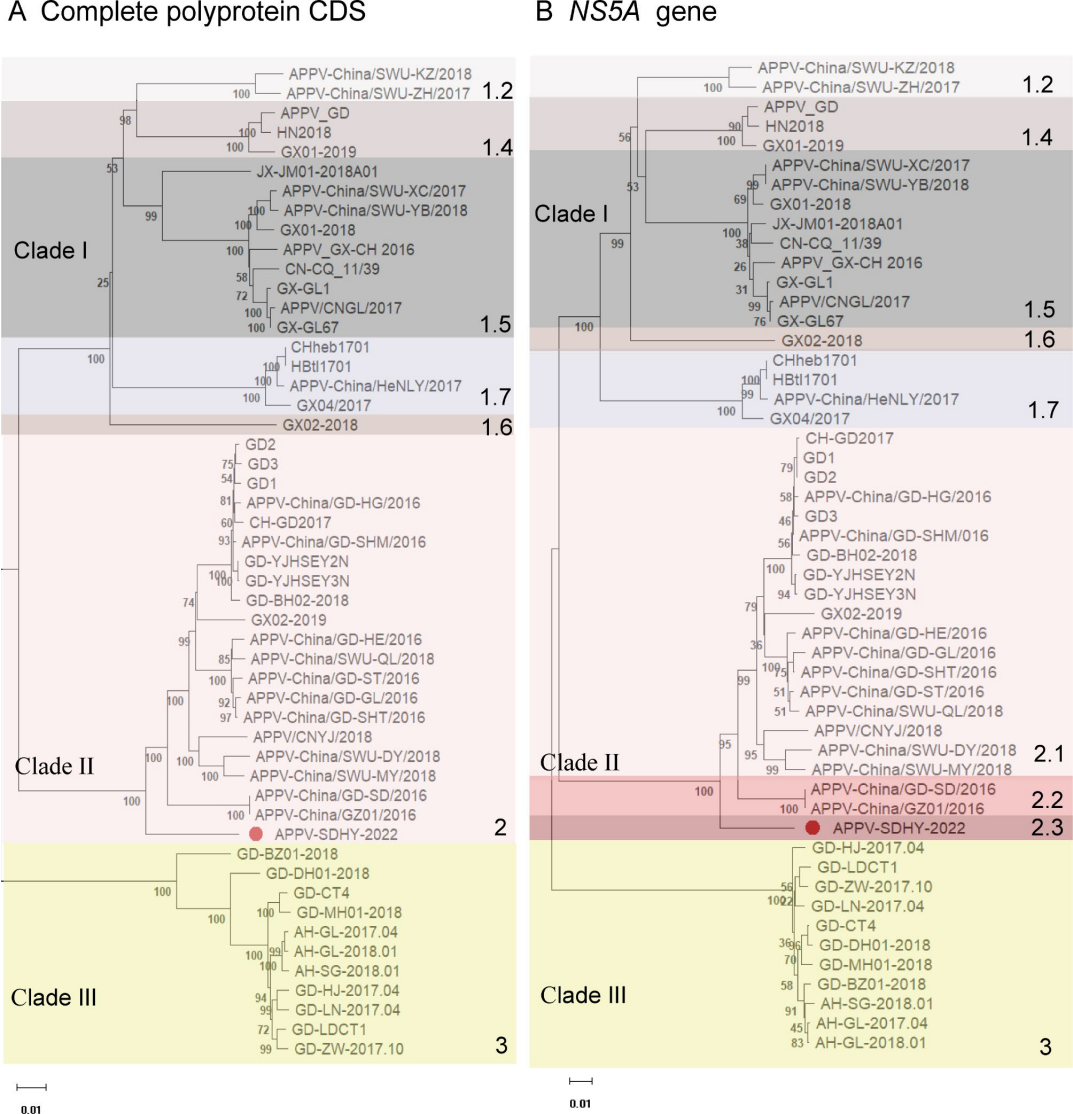
Phylogenetic analysis of Chinese APPV strains. Phylogenetic trees based on the nucleotide sequences of the complete polyprotein CDS (**A**) and the *NS5A* gene (**B**) were constructed by the neighbor-joining (NJ) method with 1,000 bootstrap replicates in MEGA11 software. The APPV-SDHY-2022 strain reported in this study is indicated with a red dot

Phylogenetic analysis was performed based on complete polyprotein CDS and NS5A nucleotide sequences. The results showed that APPV-SDHY-2022 belongs to a separate branch of Clade II (Fig. 2A). Moreover, the results revealed that the homology of NS5A nucleotide sequences was above 94.6% for the same isoform, 84.7-94.5% for different isoforms of the same clade and 76.8-81.1% for different clades (Table 3). Therefore, we proposed that Clade II strains can be further divided into three subclades and that APPV-SDHY-2022 belongs to subclade 2.3. APPV-China/GD-SD/2016 and APPV-China/GZ01/2016 belong to subclade 2.2, and the other Chinese strains among the Clade II cluster belong to subclade 2.1 (Fig. 2B). Since Clade II strains were found only in China, this typing method can help us better analyze the evolution of Clade II strains.

**Table 3:** Homology analysis of NS5A nucleotide sequence within clades or subclades (%)

Identity /Divergence	1.2	1.4	1.5	1.6	1.7	2.1	2.2	2.3	3
1.2	97.7 /2.3	89.1–89.3	88.7–89.3	87.6–88.1	85-85.3	79.2–80.9	81-81.1	80.4–80.6	79.7–80.2
1.4	10.7–10.9	98.4–99 /1-1.6	88.9–90	88.1–88.3	84.9–85.6	79.3–80.6	80.6	80.4–80.6	79.1–79.9
1.5	10.7–11.3	10-11.1	97.5–99 /1-2.5	86.8–87.6	84.7–86.7	78.7–80.2	79.3–80.7	79.1–80	78.7–79.7
1.6	11.9–12.4	11.7–11.9	22.4–23.2	100 /0	84.7–85	79-79.7	79.3	80	78.7–79.4
1.7	14.7–15	14.4–15.1	23.3–25.3	15-15.3	97.8–100 /0-2.2	78.9–80.5	79.2–79.4	79.3–80.1	77.1–78.7
2.1	19.1–20.8	19.4–21.3	19.8–21.3	20.3–21	19.5–21.1	94.6–99.9 /0.1–5.4	93.6–94.5	92.4–93.3	76.8–77.9
2.2	18.9–19	19.4	19.3–20.7	20.7	20.6–20.8	5.5–6.4	100 /0	92.7	77.5–77.9
2.3	19.4–19.6	19.4–19.6	20-20.9	20	19.9–20.7	6.7–7.6	7.3	100 /0	77.6–78.3
3	19.8–20.3	20.1–20.9	20.3–21.3	20.6–21.3	21.3–22.9	22.1–23.2	22.1–22.5	21.7–22.4	98.2–99.8 /0.2–1.8

### Recombination analysis

**Fig. 3:**
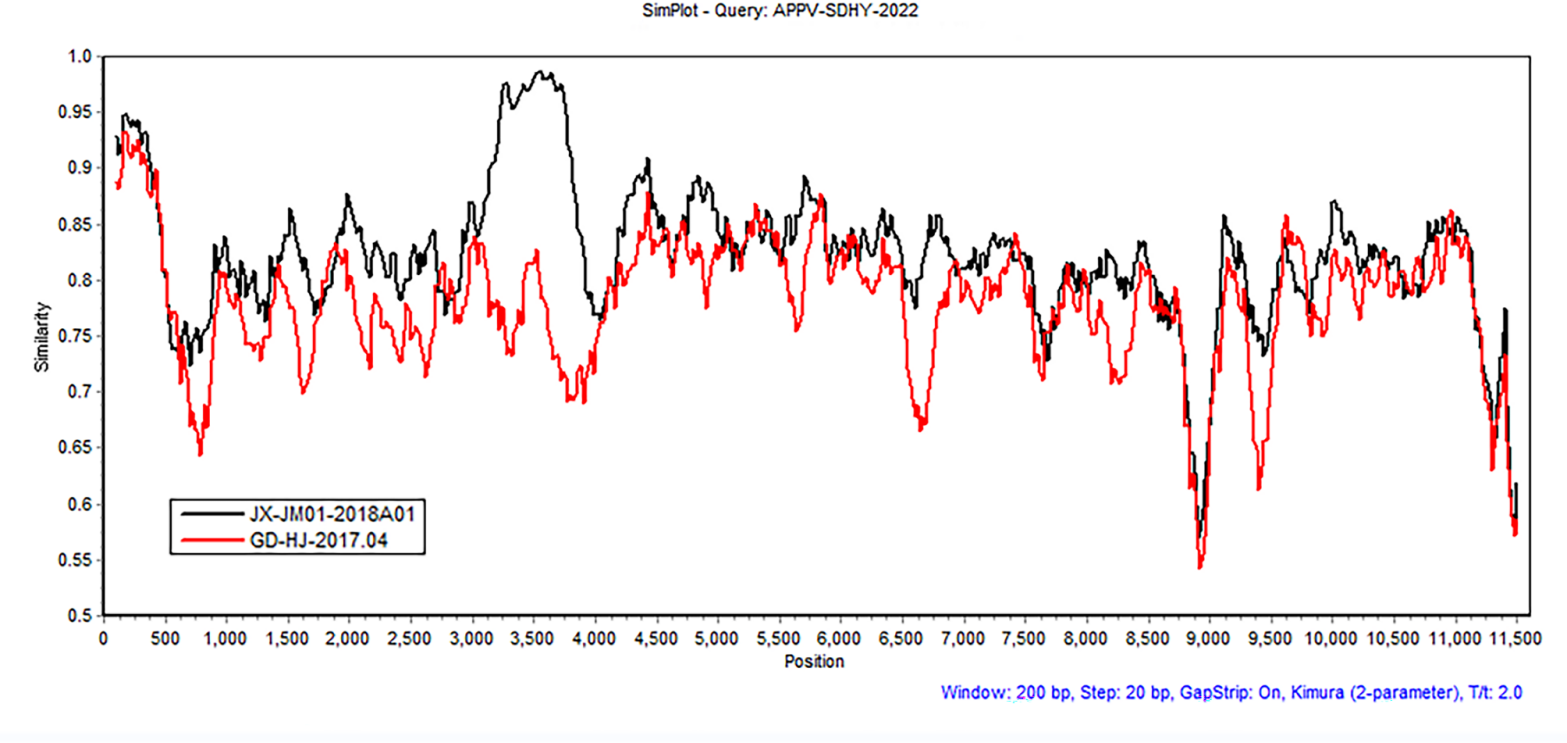
Recombination analysis of the complete genomes of the APPV-SDHY-2022 strain from Shandong Province. Potential recombination events were identified using Recombination Detection Program 4 (RDP4) and then examined using similarity plots and bootstrap analysis in Simplot 3.5.1. The major and minor parents were JX-JM01-2018A01 and GD-HJ-2017.04, respectively

To further explore the genetic evolution of APPV, potential recombination events were identified using Recombination Detection Program version 4 (RDP4) and then examined using SimPlot version 3.5.1. Among all available APPV strains, 8 strains (GD-DH01-2018, GD-BZ01-2018, JX-JM01-2018A01, GD2, GD-HJ-2017.04, GD-LN-2017.04, GD-CT4, and GD-MH01-2018) had potential genetic recombination events. Although NGS of APPV-SDHY-2022 confirmed recombination events of JX-JM01-2018A01 and GD-HJ-2017.04 by RDP4 (see Additional file 4: Table s4), no obvious genetic recombination in APPV-SDHY-2022 strains was observed by SimPlot software in this study (Fig. 3).

### Amino acid sequence analysis

**Fig. 4:**
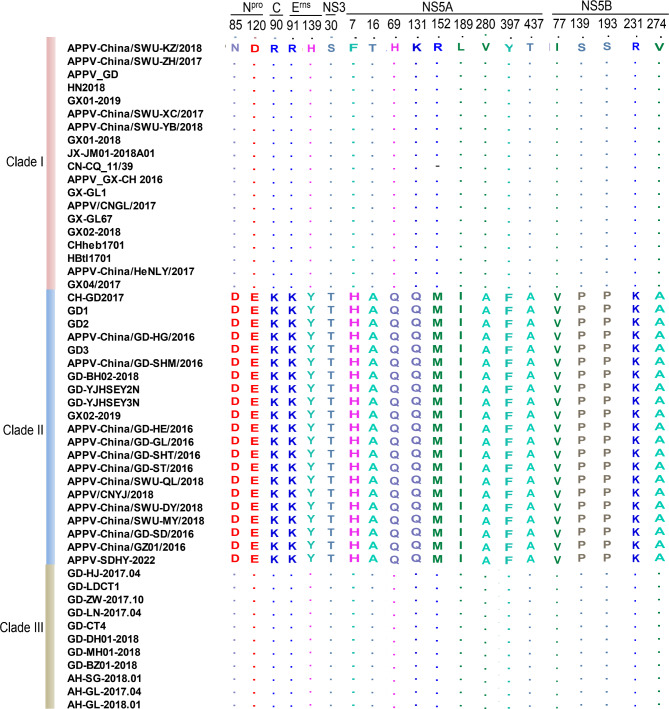
The unique amino acids found in Clade II APPV strains. Amino acid sequences of viral proteins were aligned with reference strains using MEGA11 and BioEdit software

Amino acid sequences of individual viral proteins of all the Chinese APPV strains were analyzed. No amino acid insertions or deletions were found in the APPV-SDHY-2022 strain. The amino acid sequences of the individual proteins were compared to identify those that differentiate Clade II from Clade I and Clade III, and 20 unique amino acids were found in Clade II strains (Fig. 4), among which, most sites were distributed on NS5A(7H,16A,69Q,131Q,152M,189I,280A,397F,437A) and NS5B(77V,139P,193P,231K,274A), and the remaining sites were on N^pro^ (85D,120E), C(90K), E^rns^(91K,139Y) and NS3(30T). Interestingly, the amino acids at these unique sites were identical between Clade I and Clade III strains, demonstrating that it is possible to determine the type of strain by measuring these specific amino acids alone.

### Glycosylation analysis

**Fig. 5:**
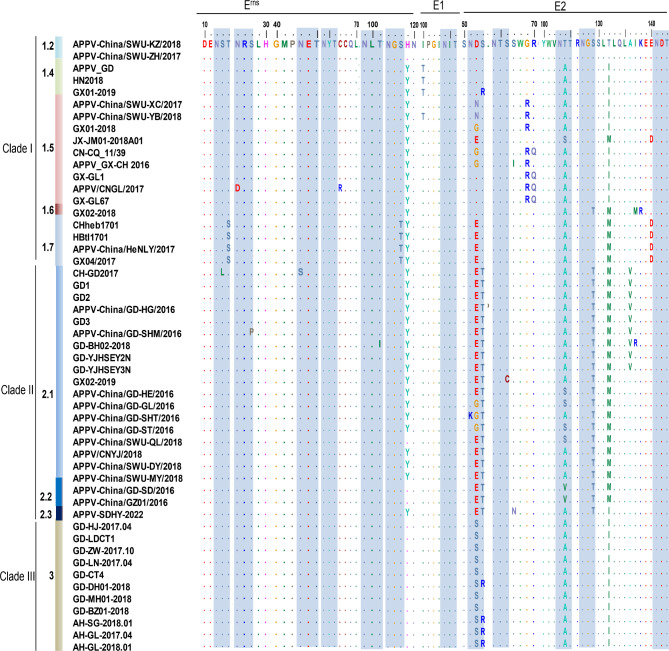
Putative N-glycosylation sites of E^rns^, E1 and E2 proteins. The putative N-glycosylation sites within the E^rns^, E1 and E2 sequences of Chinese APPV strains were predicted according to a glycosylation analysis algorithm, and are shown as a blue shaded box

In this study, putative N-glycosylation sites in the three important glycoproteins, E^rns^, E1, and E2, in Chinese APPV strains were also predicted. APPV-SDHY-2022, along with most of the strains in Clade II, is heavily glycosylated, with a total of ten N-glycosylation sites (N104 in the E1 protein; N12, N26, N43, N64, and N99 in the E^rns^ protein; N51,N64,N103, and N127 in the E2 protein) (Fig. 5). All the Chinese APPV strains had a conserved putative N-glycosylation site at N104 with a consensus N-I-T motif in the E1 protein. The putative N-glycosylation sites in the E^rns^ and E2 proteins differed greatly among strains in different subclades, and 9 patterns of putative N-glycosylation sites were observed in E2 proteins, including N51 + N64 + N103, N64 + N103, N51 + N64 + N103 + N141, N51 + N64 + N127 + N103 + N141, N51 + N64 + N103 + N127, N64 + N103 + N127, N51 + N127, N51 + N64, N64 (Fig. 5). Among the N-glycosylation sites of E2 proteins, a putative site at N64 was highly conserved.

### Antigen prediction

**Fig. 6:**
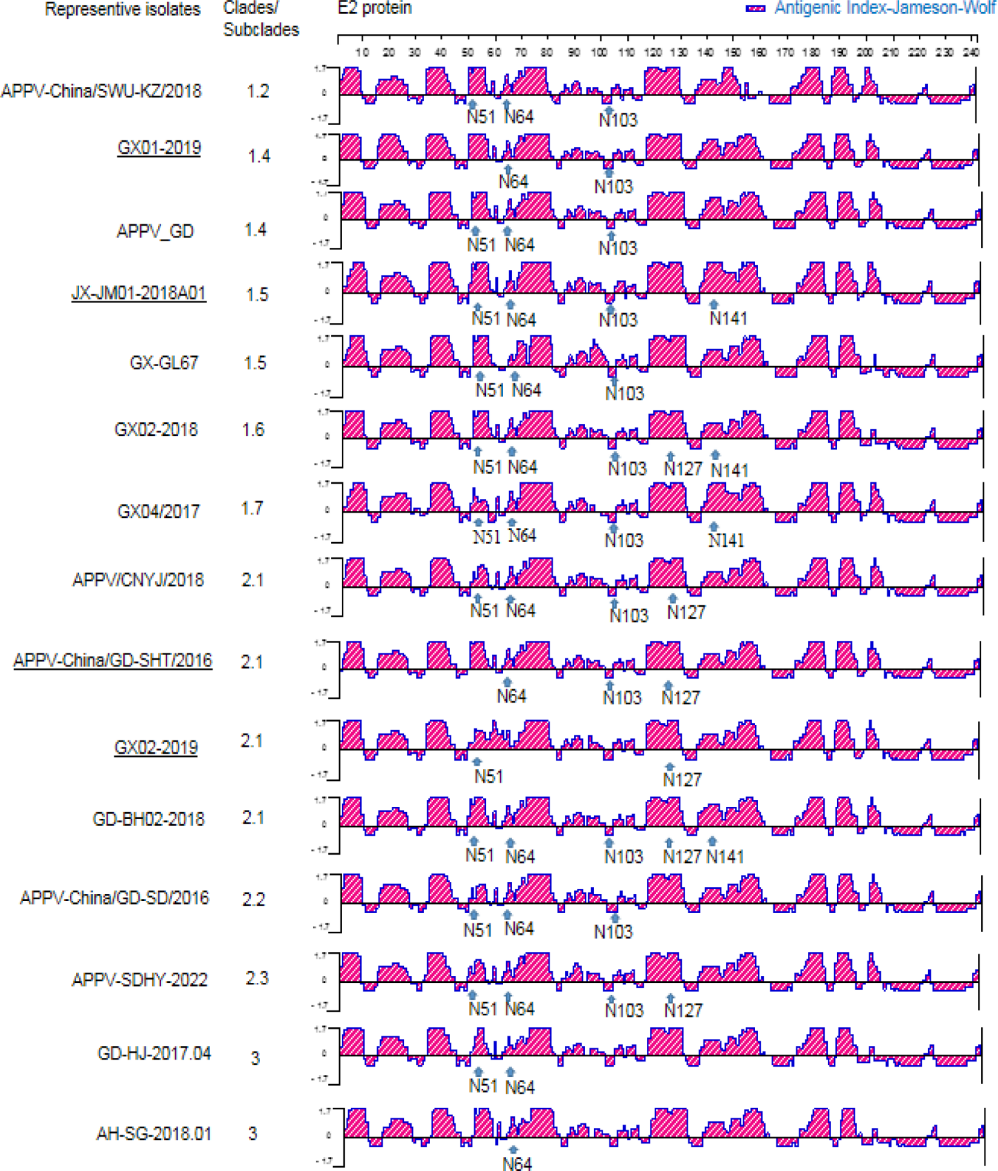
Antigenicity prediction for the E2 protein. The Jameson-Wolf algorithm, which combines secondary structure information with backbone flexibility to predict surface accessibility, was used to determine the predicted antigenic index, with a threshold value of 1.7. The putative N-glycosylation sites within the E2 sequences of Chinese APPV strains are shown as a blue arrow. Representative strains from different Clades/subclades or patterns of putative N-glycosylation sites were included, and the strains in each subclade with different patterns of putative N-glycosylation sites are underlined

To analyze the effect of glycosylation sites on the antigenicity of the E2 protein, the antigenic index was determined by the Jameson-Wolf method in this study, and the results showed that aa positions at 1 ~ 9, 15 ~ 28, 34 ~ 44, 49 ~ 55, 62 ~ 82, 118 ~ 130, 136 ~ 158, 174 ~ 184, 188 ~ 196 and 200 ~ 205 of the E2 protein were the potential immunodominant regions. A comparison of the antigenic index within Chinese strains with and without a specific putative site showed that the putative N-glycosylation site at N51 had a negative effect on the antigenicity of the corresponding region (Fig. 6).

**Fig. 7:**
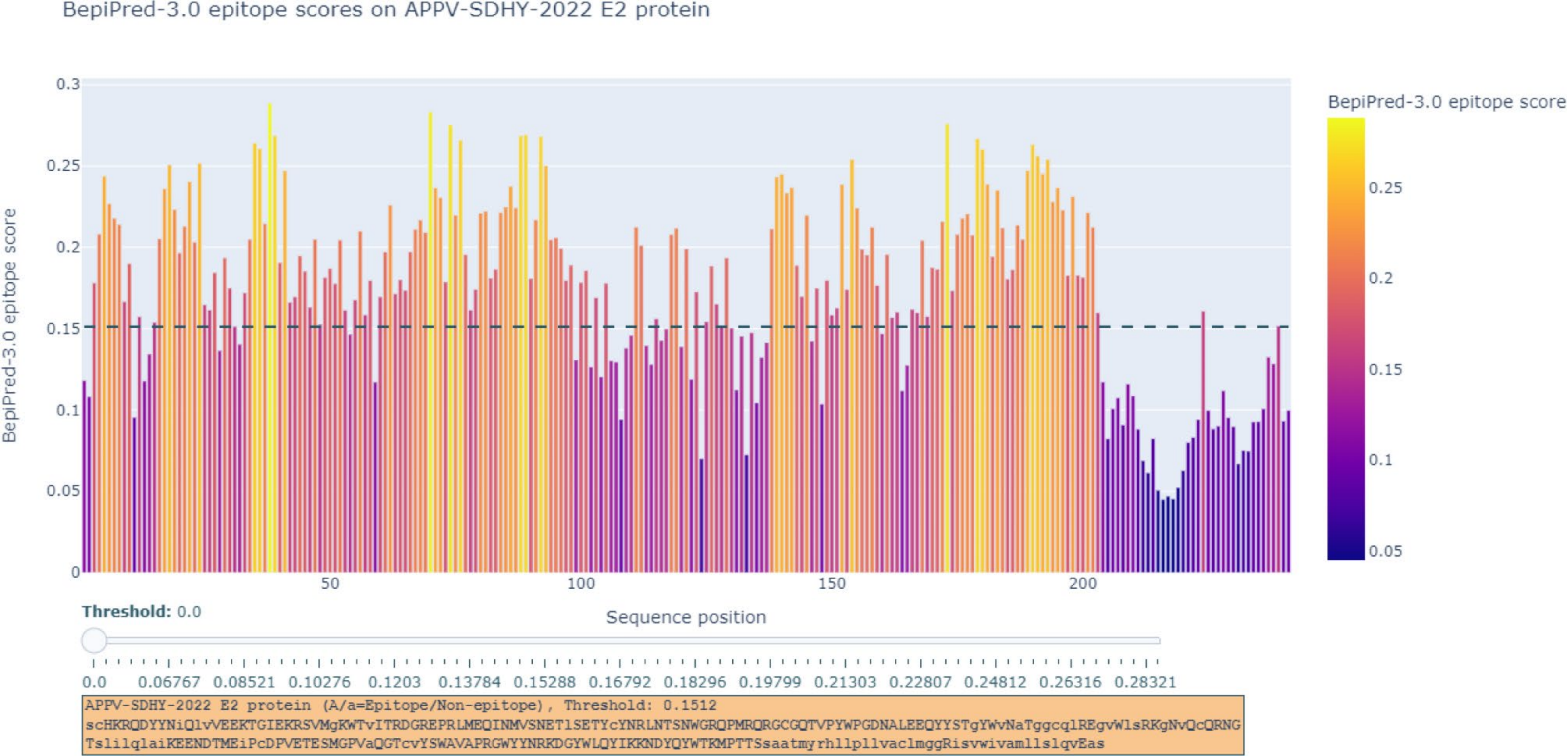
Conformational B-cell epitope prediction for the E2 protein. The potential B-cell conformational epitopes of the E2 protein in APPV Chinese strains were predicted by BepiPred-3.0, and the residues with scores above the threshold (default value is 0.1512) are predicted to be part of an epitope and colored in yellow on the graph (where Y-axes depict BepiPred-3.0 epitope scores and X-axes protein sequence positions). Shown is the graphical output of B-cell discontinuous epitope predictions for the E2 protein with APPV-SDHY-2022 as an example

To further analyze the effect of glycosylation sites on conformational epitopes of the E2 protein, BepiPred-3.0 was used to predict B-cell conformational epitopes. The results showed that the 15 most likely B-cell conformational epitope residues varied among different Clades/subclades or patterns of N-glycosylation sites, and 39E, 70R, 173R, 190K, and 191N were conserved residues among all Chinese strains (Table 4) (see also the graphical representations of the predicted epitopes in Fig. 7).

**Table 4:** Prediction of potential B-cell conformational epitopes from E2 protein sequence

Representive isolates	Clades/ Subclades	Patterns of putative N-glycosylation sites	TOP 15 most likely B-cell epitops residues
APPV-China/SWU-KZ/2018	1.2	N51 + N64 + N103	5R, 22E, 24R, 35R, 36E, 38R, 39E, 70R, 74R, 76R, 173R, 190K, 191N, 192D, 193Y
GX01-2019	1.4	N64 + N103	18K, 35R, 36E, 38Q, 39E, 53R, 70R, 74R, 76R, 173R, 179R, 190K, 191N, 192D, 193Y
APPV_GD	1.4	N51 + N64 + N103	22E, 24R, 35R, 36E, 38R, 39E, 70R, 74R, 76R, 173R, 179R, 190K, 191N, 192D, 193Y
JX-JM01-2018A01	1.5	N51 + N64 + N103 + N141	24R, 35R, 36E, 38R, 39E, 70R, 74R, 76R, 173R, 179K, 180K, 190K, 191N, 192D, 193Y
GX-GL67	1.5	N51 + N64 + N103	22E, 24R, 35R, 36E, 38R, 39E, 70R, 74R, 76R, 142D, 152E, 173R, 179R, 190K, 191N
GX02-2018	1.6	N51 + N64 + N127 + N103 + N141	22E, 24R, 35R, 36E, 38R, 39E, 70R, 74R, 76R, 173R, 179R, 190K, 191N, 192D, 193Y
GX04/2017	1.7	N51 + N64 + N103 + N141	22E, 35R, 36E, 38R, 39E, 70R, 71R, 74R, 76R, 173R, 179R, 180K, 190K, 191N, 192D
APPV/CNYJ/2018	2.1	N51 + N64 + N103 + N127	24R, 35R, 36E, 38R, 39E, 70R, 74R, 76R, 88D, 173R, 179R, 180K, 190K, 191N, 193Y
APPV-China/GD-SHT/2016	2.1	N64 + N103 + N127	22E, 24R, 35R, 36E, 38R, 39E, 41R, 70R, 74R, 76R, 154E, 173R, 179R, 180K, 190K
GX02-2019	2.1	N51 + N127	5R, 35R, 36E, 38R, 39E, 62R, 70R, 74R, 76K, 88D, 173R, 183Y, 190K, 191N, 193Y
GD-BH02-2018	2.1	N51 + N64 + N127 + N103 + N141	35R, 36E, 38R, 39E, 70R, 74R, 139E, 173R, 179K, 180K, 181D, 183Y, 189K, 190K, 191N
APPV-China/GD-SD/2016	2.2	N51 + N64 + N103	18K, 35R, 36E, 38R, 39E, 70R, 74R, 76R, 173R, 179R, 180K, 190K, 191N, 192D, 193Y
APPV-SDHY-2022	2.3	N51 + N64 + N103 + N127	35R, 36D, 38R, 39E, 70R, 74R, 76R, 88D, 89N, 92E, 173R, 179R, 180K, 190K, 191N
GD-HJ-2017.04	3	N51 + N64	17E, 36E, 38Q, 39E, 70R, 76R, 139E, 173R, 179R, 180K, 189K, 190K, 191N, 192D, 193Y
AH-SG-2018.01	3	N64	18K, 36E, 38Q, 39E, 70R, 74K, 76R, 88D, 173R, 179R, 180K, 189K, 190K, 191N, 193Y

## Discussion

One phylogeographic analysis suggested that the APPV population possibly originated in the Netherlands and was introduced into China between 1837 and 2010 [23]. Guangdong is the main node of transmission, and the pattern of transmission of the Chinese lineage shows a trend of movement from south to north [23]. Thus, relatively few virulent strains have been found in northern China compared to southern China. To date, in northern China, only two strains, HBtl1701 and CHheb1701, from Hebei Province are available with complete CDSs in GenBank. The APPV-SDHY-2022 strain in this study is the first complete genome of APPV in Shandong Province of northern China, and our results contribute to a better understanding of APPV epidemiology in China.

Previously, most samples used for APPV detection were from CT cases with tissues (spleens, lymph nodes, etc.) or serum samples [4, 7, 9, 11–14, 17–21, 24], while detection in placentas and umbilical cords samples is lacking. In this study, APPV was detected for the first time in the placenta, umbilical cords and aborted fetus of abortion cases. After infection of the pregnant sow, the virus may have crossed the placental barrier and infected the fetus, and abortion occurred at this time. Previous reports showed by experimental inoculation that APPV can be vertically transmitted by transplacental infection [25, 26], and all sows inoculated with the APPV far-rowed piglets were affected by CT; however, in our study, the surviving piglet showed no signs of CT. Among other identified viruses, porcine picobirnavirus, porcine kobuvirus, porcine sapovirus, Po-Circo-like virus, porcine serum-associated circular virus and porcine bocavirus 1 were present in healthy and diarrheic pigs [27, 28], Getah virus, porcine parvovirus 1, porcine parvovirus 5 and porcine circovirus 3 may contribute to abortion [29–31], however, considering the read counts and in-depth analyses, APPV was the dominant coexistence virus. Meanwhile, no contigs were identified with other known abortion-related pathogens (PRRSV, PPV2-4/6–8, CSFV, PCV2 and Japanese encephalitis virus), which infers that APPV may be related to abortion. However, APPV can be detected in healthy pigs [21], and an APPV detection rate of 2.4% was reported in apparently healthy pigs in Germany [32]; thus, whether APPV contributes to abortion needs further research and verification.

Although the variability and evolution of APPV have been investigated previously, in-depth and systematic investigations are still needed [8]. In this study, genetic evolutionary analysis revealed that APPV-SDHY-2022 belongs to Clade II but is not on the same branch as the other strains in Clade II. One study showed that of all the genes in the virus, the NS5A coding sequence was the most suitable choice for identifying the APPV strain, as it was able to reproduce the same phylogenetic and evolutionary information as the entire viral genome [33]. Therefore, we examined the homology of NS5A nucleotide sequences, and we found that Clade II could further be subtyped and classified APPV-SDHY-2022 as subclade 2.3. This result indicated that there were highly variable regions in the whole genomic sequences, which would be a major challenge for molecular diagnosis and epidemiological investigation of APPV, and the conserved 5’ UTR regions may be a more ideal target for molecular detection [34].

A previous study suggested that recombination events occur between clades (Clades II and III) or within a clade (Clade I) [13]. In our recombination analysis of 51 APPV strains, no recombination event was observed in the APPV-SDHY-2022 strain from Shandong Province, and all eight recombinant strains were isolated in other provinces (Guangdong and Jiangxi) in China. For the amino acid analysis, no amino acid insertions or deletions were found within APPV-SDHY-2022 strain; interestingly, we found numerous amino acids specific to Clade II. By identifying specific amino acids, it is possible to determine the genotype of a virus strain, providing a novel approach for the rapid detection of virus genotypes.

Putative N-glycosylation sites in the three important glycoproteins in Chinese APPV strains were also predicted, since the glycosylation status of the pestivirus glycoproteins plays an important role in virulence [35]. The APPV-SDHY-2022 strain is heavily glycosylated and has ten N-glycosylation sites, similar to most of the strains in Clade II (APPV-China/SWU-DY/2018 or APPV/CNYJ/2018) [6]. For some viruses, such as PRRSV, N-glycosylation sites are related to vaccine protection. In APPV, the N-glycosylation sites in the three important glycoproteins may also have the same important biological characteristics, and the E2 protein exhibited the greatest diversity of N-glycosylation sites, which would create a major bottleneck in APPV vaccine design, since the E2 protein is the main immunogenic protein and the crucial target for APPV vaccine development [36, 37]. Our results showed 5 conserved residues (39E, 70R, 173R, 190K, 191N) of B-cell conformational epitopes among all Chinese strains, which could be targets for multiepitope subunit vaccine and monoclonal antibody preparation, since BepiPred-3.0 is trained on PDB crystal structures of ab-ag complexes, and to predict antigen residues that are in contact with an antibody [22].

APPV can infect both domestic pigs and wild boar populations via horizontal and vertical transmission, which can result in the loss of piglets and decrease in pig reproductive performance [38]. Due to the abovementioned complicated epidemiology and the enormous loss of infected swine herds, epidemiological research should be thoroughly continued, and vaccine research should be accelerated.

## Conclusions

In summary, in this study, APPV was detected in placenta, umbilical cords and aborted piglet samples from abortion cases using viral metagenomic sequencing, and this is the first time that the whole genome of APPV has been detected in Shandong Province. As a result of the analysis, new typing methods and genotype detection methods have been proposed, which form the basis for future related research.

### Electronic supplementary material

Below is the link to the electronic supplementary material.

**Additional file 1: Table s1**. Information on the Chinese reference strains used in this study

**Additional file 2: Table s2**. Blast results of assembled sequences for RNA sample (exclusion of phages and sequences with alignment length less than 100 bp). **Table s3**. Blast results of assembled sequences for DNA sample (exclusion of phages and sequences with alignment length less than 100 bp)

**Additional file 3: Fig. s1**. APPV confirmation by NS3 gene RT–PCR (A) and sequencing (B). The sample pool was APPV positive by RT–PCR amplification targeting to the NS3 gene (lane 1 and lane 2). The assembled sequence of the PCR products had 100% identity with that of APPV-SDHY-2022

**Additional file 4: Table s4**. The recombination events detected by RDP4.0 after complete genome sequence alignment of all APPV Chinese strains

## Data Availability

The data used and analyzed during the current study are available from the corresponding author upon reasonable request.

## Abbreviations

APPV: Atypical porcine pestivirus
CDS: Complete coding sequence
CSFV: Classical swine fever virus
CT: Congenital tremor
NGS: Next-generation sequencing
ORF: Open reading frame
PCV: Porcine circovirus
PPV: Porcine parvovirus
PRRSV: Porcine reproductive and respiratory syndrome virus
qRT–PCR: Quantitative RT–PCR
UTR: Untranslated region
