# Supplementary figures and images for: Genome characteristics of atypical porcine pestivirus from abortion cases in Shandong Province, China
Source: Virol J. 2023 Nov 29;20:282. doi: 10.1186/s12985-023-02247-0 (PMC10688472; doi:10.1186/s12985-023-02247-0)

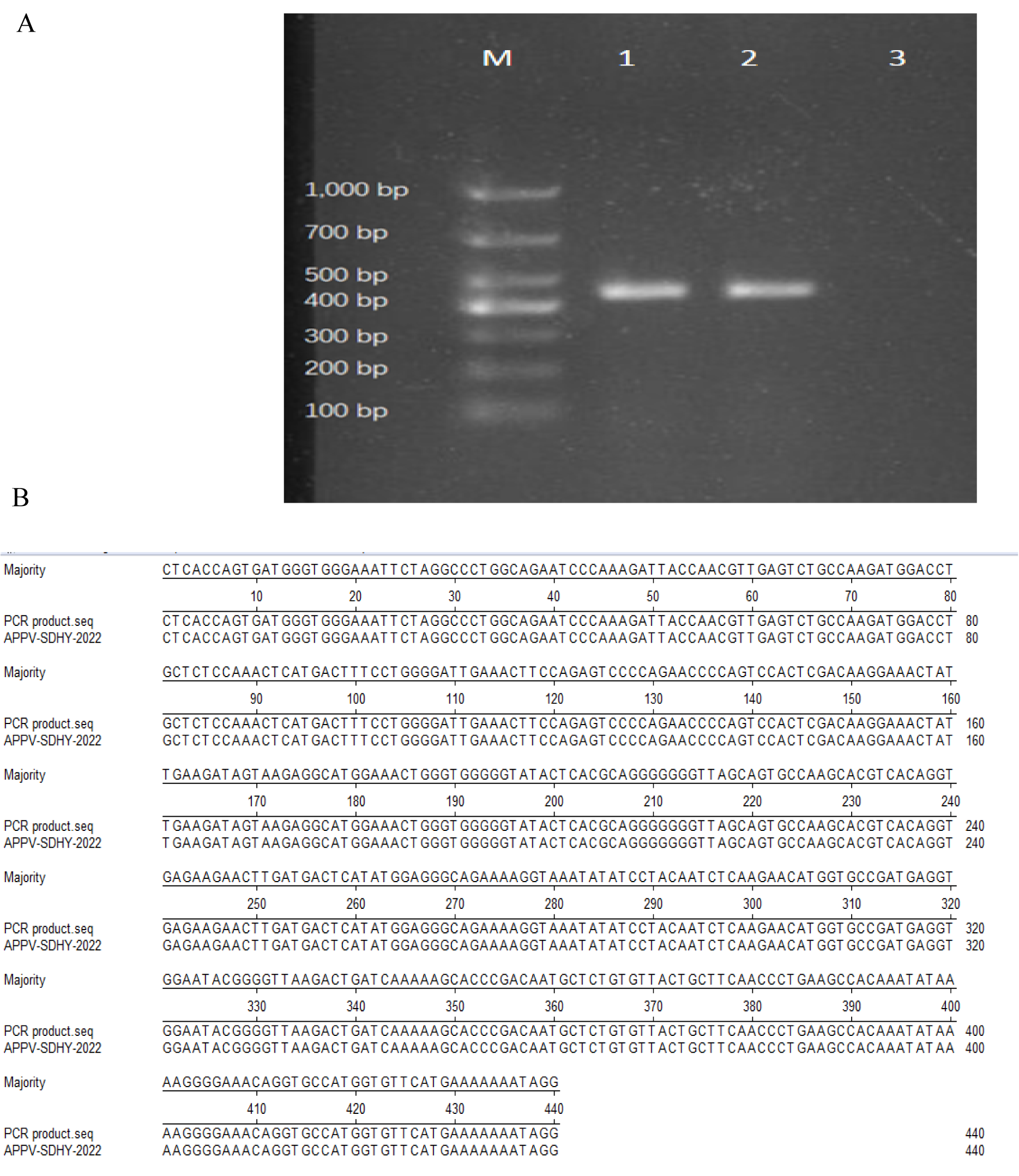

Supplement: Supplementary file 3 — Additional file 3: Fig. s1. APPV confirmation by NS3 gene RT–PCR (A) and sequencing (B). The sample pool was APPV positive by RT–PCR amplification targeting to the NS3 gene (lane 1 and lane 2). The assembled sequence of the PCR products had 100% identity with that of APPV-SDHY-2022 [file 12985_2023_2247_MOESM3_ESM.png]
